# Structural and functional abnormalities of the tongue: An epidemiological study from a tertiary care center in India

**DOI:** 10.12688/f1000research.131661.2

**Published:** 2024-02-16

**Authors:** Angela Piplani, Mathangi Kumar, Ravindranath Vineetha, Ranjanee Srinivasan, Kalyana Chakravarthy Pentapati

**Affiliations:** 1 Queen Mary University of London, London, UK; 2 Department of Oral Medicine & Radiology, Manipal College of Dental Sciences, Manipal, Manipal Academy of Higher Education, Manipal Academy of Higher Education, Manipal, Karnataka, 576104, India; 3 Department of Public Health Dentistry, Manipal College of Dental Sciences, Manipal, Manipal Academy of Higher Education, Manipal Academy of Higher Education, Manipal, Karnataka, 576104, India

**Keywords:** Tongue Disease, Glossitis, Prevalence Study, Stomatognathic System Abnormalities

## Abstract

**Background:**

The tongue is a prominent muscular organ of the oral cavity and the integrity of the tongue mucosa frequently can reflect the overall health of an individual. Many notable structural and functional alterations can affect the tongue. These changes may be symptomatic or asymptomatic. Hence, the aim of the study was to determine the prevalence of structural and functional abnormalities of the tongue in a population reporting to a tertiary care center. We also assessed the prevalence of the normal variants and evaluated the normal tongue protrusion measurement in the study population.

**Methods:**

The cross-sectional study included 1,143 dental outpatients above 18 years of age who visited the Department of Oral Medicine and Radiology between October 2021 to February 2022. Demographic details of the patients were noted. Participants were asked questions regarding any symptoms or abnormalities noticed on the tongue. The tongue was examined thoroughly for any structural/ functional abnormalities. The maximal tongue protrusion for each participant was measured by asking them to extend their tongue out. Medical history, drug history, and social history were recorded.

**Results:**

The study included 564 male and 579 female participants; tongue lesions were positive in 66.5% of the study population. Coated tongue (26.2%) was the most frequent structural abnormality that was noted in the present study. Taste dysfunction (4.6%) was the most frequent functional abnormality. The lesions were mostly in the anterior two-thirds (4.2%) of the tongue. Functional abnormalities of the tongue were noted in 8.7% of the study participants.

**Conclusions:**

The results of the present study showed that structural and functional abnormalities of the tongue were present in 66.5% of the population. Careful and detailed evaluation of the tongue examination is mandated in routine dental checkups. This shall help in the prompt identification of various etiological factors causing structural and functional abnormalities of the tongue.

## Introduction

The tongue is a prominent muscular organ of the oral cavity that plays a vital role in speech, mastication, and digestion. The integrity of the tongue mucosa frequently reflects the oral and systemic health of an individual. ^1^ The oral cavity, being the portal of entry to various infectious and carcinogenic agents, mucosa of the tongue is easily exposed to pathologic changes. ^2^ Numerous oral and systemic diseases affect the tongue, impairing its structure and function, and owing to its strategic position, those tongue changes can be easily appreciated. Notable structural alterations including the color, texture, size, shape, and appearance of the tongue can occur due to a variety of local causes or systemic conditions. Functional abnormalities of the tongue include inability/restriction of the tongue movements, taste disturbances, and/or discomfort. This can often occur in neurological conditions (palsy), oral submucous fibrosis, malignancy affecting the base of the tongue, neuromuscular disorders, and post-radiotherapy. The tongue changes can either be due to local factors like trauma, contact allergy, benign, reactive, or malignant growth or due to underlying systemic conditions like anemia nutritional deficiencies, infections, hormonal disturbances, immune-mediated diseases, and metabolic conditions. ^3^ Even though tongue manifestations can be the early and easily identifiable diagnostic clue, it is often overlooked during a dental examination unless the patients report specific tongue complaints. Various studies have assessed the prevalence of tongue abnormalities in different population groups, however, the clinical correlation with the changes noted in the tongue is seldom looked into. ^1^^,^^3^ Few recent prevalence studies have focused on the structural abnormalities of the tongue in pediatric and geriatric populations. ^4^^–^^6^ However, we could not find population-specific studies that have assessed both the structural and functional abnormalities of the tongue. The present study intended to assess the prevalence of structural and functional abnormalities of the tongue in patients visiting a tertiary care hospital and its possible association with the patient’s history and the symptoms reported. This study will help in the understanding of the commonly encountered symptomatic and asymptomatic tongue abnormalities in patients. The present study also assessed the physiological range of tongue protrusion in the study population as such data is unavailable in the literature.

## Methods

This cross-sectional study included the outpatients visiting the various units of Kasturba Medical College & Hospital and the Dental outpatient department of Manipal College of Dental Sciences, Manipal for 20 weeks from September 1 ^st^, 2021. Kasturba Medical College and Kasturba Hospital Institutional Ethics Committee approved the study protocol (IEC 409/2021 approved on 8 ^th^ August 2021). This project was registered with the Clinical Trials Registry - India (CTRI/2021/09/036996).

The inclusion criteria were all patients above 18 years of age (Indian origin) who visited the dental and medical outpatient department. Patients with severe restriction in mouth opening, those who were not able to provide the consent for the study (patients with psychiatric disorders), or not willing to participate were excluded from the study. Informed written consent was obtained from individuals who were willing to participate in the study. The following data were noted down for each patient: age, and gender, and the participants were asked about any symptoms or abnormalities noticed on the tongue. Also, the medical history, drug history, and oral abusive habit history were recorded for each patient in a specially designed proforma.

### Clinical examination

The clinical examination of the oral cavity and tongue was performed following WHO guidelines (Geneva, 1997) in a well-lit, noisy, and crowd-free room on a dental chair with artificial lighting and a mouth mirror under aseptic conditions. ^7^ Three examiners independently (authors: AP, MK, RS) performed the clinical examination of the tongue for the patients and in case of doubt consensus was arrived after consultation with a clinical expert (authors RV - with over fifteen years of experience). Any structural abnormality of the tongue related to color, texture, size, shape, or appearance were noted. The tongue was checked for depapillation, coating or inflammation, any ulcers, white lesions, red patches, pigmentation, varices, and growth/swelling.

If any of the mentioned changes were observed, the location, extent, and surface characteristics of these lesions were recorded. The oral mucosal pathologies were clinically diagnosed based on their characteristic history and presenting features. The diagnosis of lichen planus was given when a bilateral white lacy pattern was observed with characteristic Wickham’s striae. When scrapable white patches were seen in medically compromised patients leaving behind erythematous surfaces on rubbing a diagnosis of candidiasis was recorded. Leukoplakia is defined as “white plaques of questionable risk having excluded (other) known that carry no increased risk for cancer. It was diagnosed by excluding other causes of raised non-scrapable white diseases or disorders patches, especially in patients with a strong habit history. Erythroplakia appeared as a raised red velvety patch that could not be attributed to any other disease. Oral submucous fibrosis is typically diagnosed in the presence of fibrotic bands and the marbled appearance of the buccal mucosa. Erythema multiforme presents as multiple oral ulcers with crusting of the lips. Allergic stomatitis is established when multiple acute onset ulcers/erythema with a history of known triggering agents. ^8^ Suspicious growth with induration of base is diagnosed as malignancy and was sent for histopathologic examination for definitive diagnosis.

**Figure 1.**
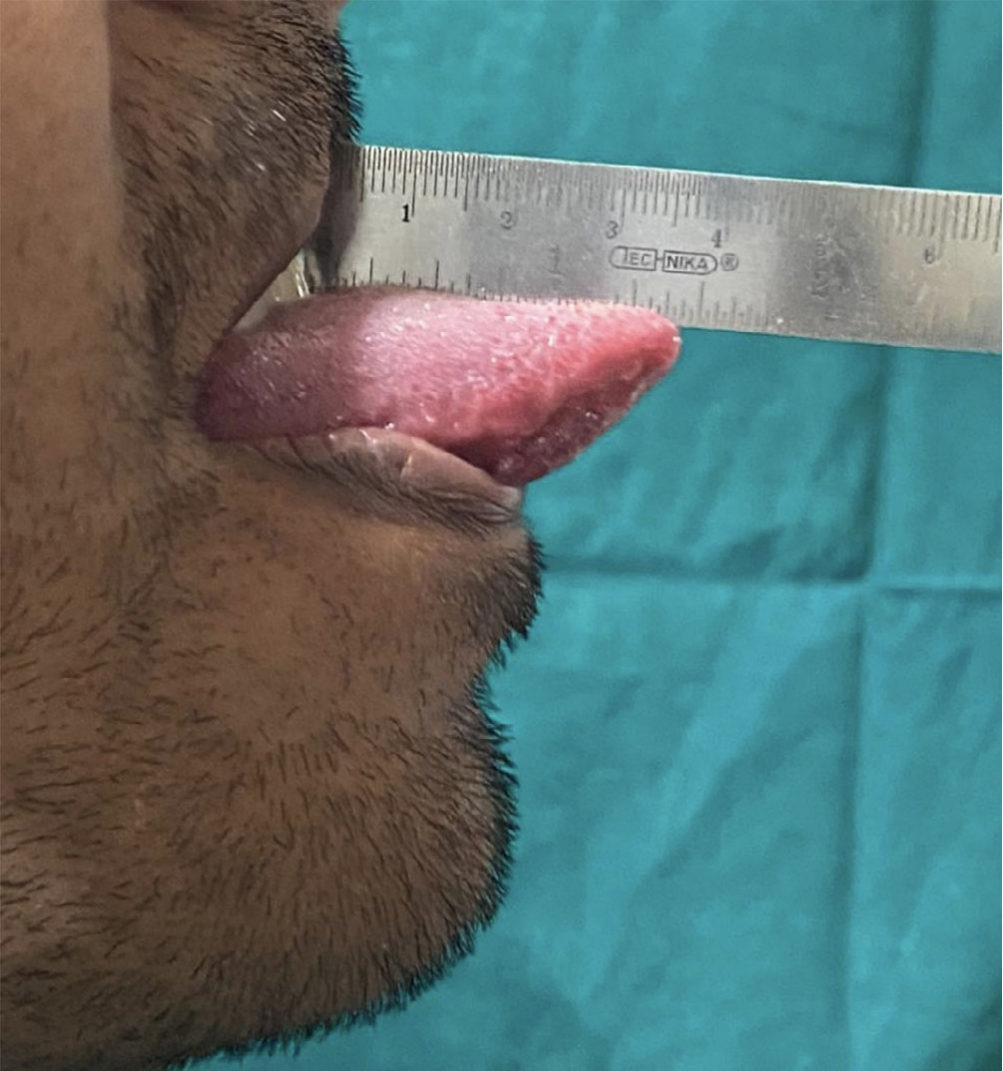
Measurement of maximum tongue protrusion.

Also, subjective symptoms and functional abnormalities like dysgeusia, burning tongue, taste loss, and tongue protrusion interferences were noted. Tongue movements were evaluated and maximal tongue protrusion was measured. This was performed by asking the patient to protrude the tongue to the maximum, and measurement was done using a sterile steel scale by measuring the distance between the maxillary incisal tip and the tip of the tongue ( Figure 1). Any deviation of the tongue during protrusion or difficulty in protrusion was also recorded.

### Data analysis

All the analysis was done using SPSS (RRID: SCR_002865) version 18 (SPSS Inc. Released 2009. PASW Statistics for Windows, Version 18.0. Chicago: SPSS Inc.). A p-value of <0.05 was considered statistically significant. Categorical variables were compared using Fisher's exact test or Chi-squared test.

## Results

The study population included 1,143 patients of which 564 were male (49.3%) and 579 were female (50.7%). The mean age of the subjects was 38.59 years old with an age range of 18–85 years old (Standard Deviation; SD=13.88). Among the various comorbidities observed, 10.5% of the study population presented with diabetes mellitus followed by hypertension (8.9%), thyroid disorders (5.0%), immunocompromised state (2.8%) and anemia (0.8%). Oral abusive habits were reported by 25.7% of the subjects. Tobacco consumption (14.6%) was more commonly reported than alcohol (11.1%). Subjective symptoms including pain (7.0%) and burning sensation (8.2%) were also reported by the study population.

Overall, 91.3% of them had a normal texture of the dorsal surface of the tongue and 7.1% had smooth bald tongue. The coral pink colored physiologic appearance was noted in 91.9%, 5.9% had a red tongue and 0.9% had a pale tongue. Figure 3 shows the topographic distribution of the tongue abnormalities observed in the study population. Figure 4 shows the various structural abnormalities observed in the present study.

The distribution of various structural tongue abnormalities is presented in Table 1. ^15^

**Table 1.** Frequency distribution of various tongue lesions.

Lesions	Number, n (%)
**Normal anatomic variations**	
Fissured tongue	13 (1.1)
Geographic tongue	16 (1.4)
**Surface alterations variations**	
Macroglossia	36 (3.1)
**Surface and color alterations**	
Depapillation	72 (6.3)
Coating on tongue	300 (26.2)
Physiologic melanin pigmentation	55 (4.8)
Varices	97 (8.5)
**Oral mucosal lesions**	
Ulcers	48 (4.2)
Candidiasis	18 (1.6)
Pemphigus	3 (0.3)
Erythroplakia	1 (0.1)
Erythema multiforme	4 (0.3)
Allergy	9 (0.8)
Leukoplakia	5 (0.4)
Lichen planus	18 (1.6)
Oral Submucous Fibrosis	29 (2.5)
**Reactive and neoplastic growths**	
Traumatic fibroma	28 (2.4)
Verrucous carcinoma	2 (0.2)
Squamous Cell Carcinoma	4 (0.3)

**Figure 2.**
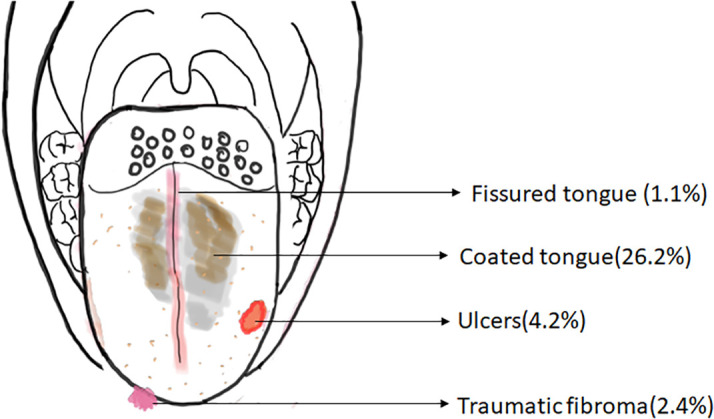
Schematic diagram depicting the distribution of the common structural abnormalities of the tongue.

The schematic representation of the common structural abnormalities of the tongue observed in the present study is shown in Figure 2.

**Figure 3.**
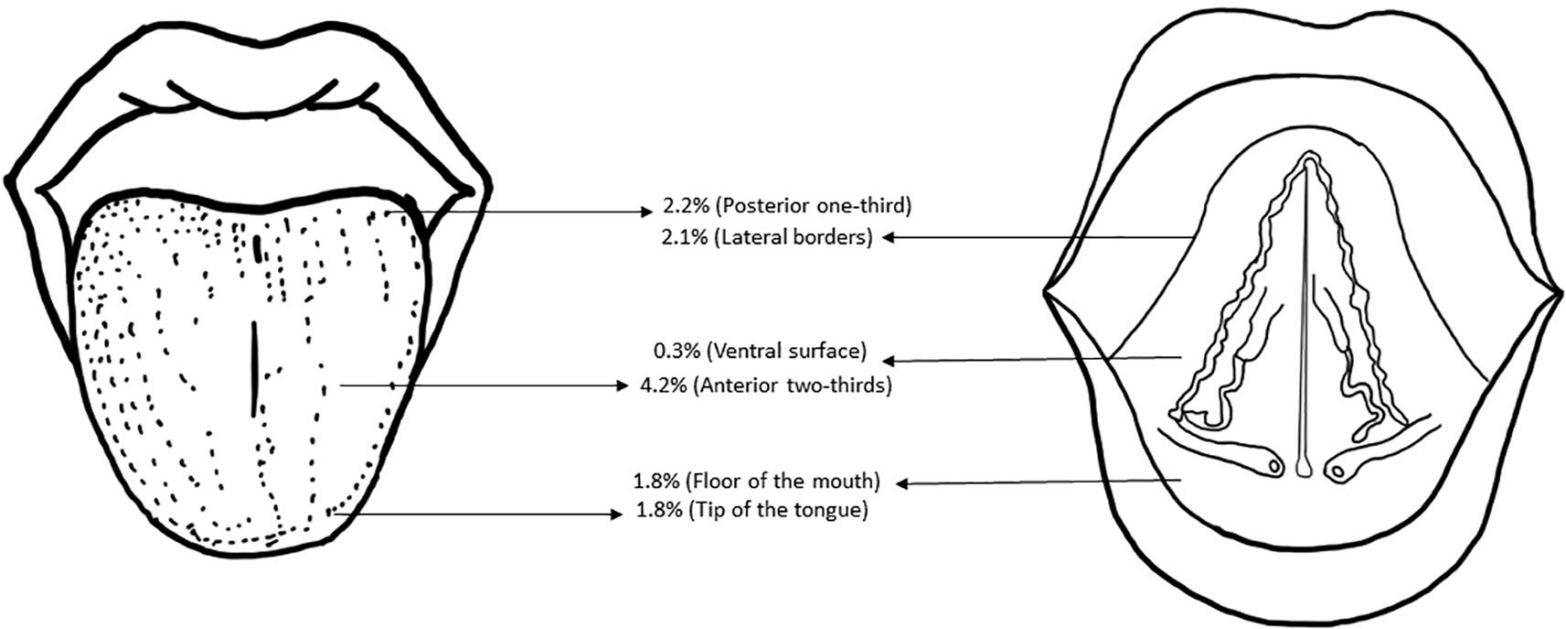
Schematic diagram depicting the topographic distribution of the tongue abnormalities in the study population.

**Figure 4.**
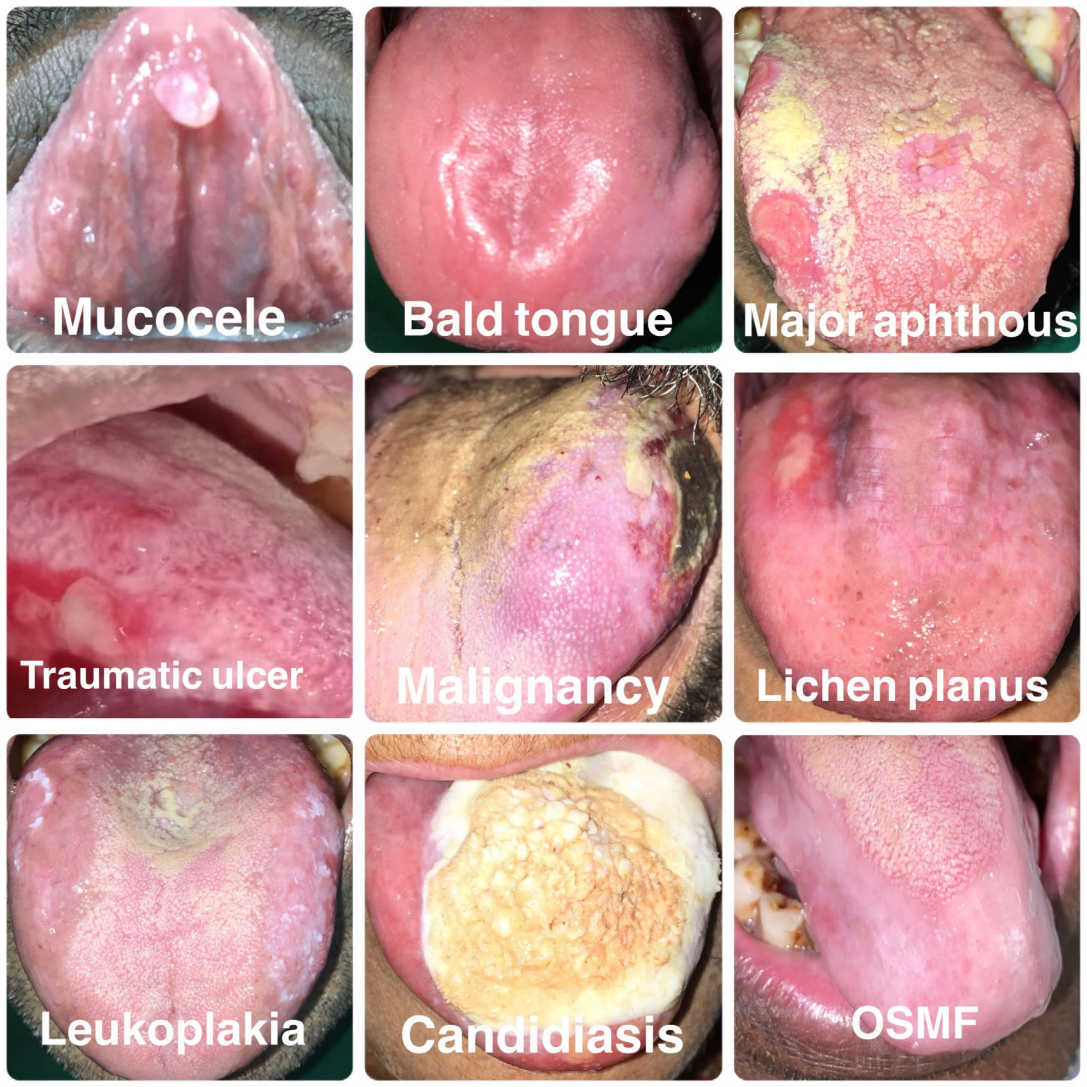
Various structural abnormalities observed in the present study.

When the site-wise distribution of structural tongue abnormalities were compared amongst the genders, males showed a statistically significant increase in the abnormalities in the anterior two-thirds of the tongue, the floor of the mouth, and the lateral border of the tongue (p=0.001, p<0.001, p<0.0001 respectively) ( Table 2).

**Table 2.** Table depicting the structural tongue abnormalities in various sites according to gender.

Site	Male n (%)	Female n (%)	p-value
Anterior 2/3 ^rd^	35 (6.2)	13 (2.2)	0.001*
Posterior 1/3 ^rd^	15 (2.7)	10 (1.7)	0.316
Floor of mouth	19 (3.4)	2 (0.3)	<0.001*
Inferior surface of the tongue	3 (0.5)	0 (0)	0.12
Tip of the tongue	11 (2)	10 (1.7)	0.828
The lateral border of the tongue	23 (4.1)	1 (0.2)	<0.001*

Also, subjects between 41 to 65 years of age showed statistically significant difference in the structural lesions occurring in the floor of the mouth and the lateral border of the tongue (p<0.001 and p=0.024 respectively) compared to the other age groups ( Table 3).

**Table 3.** Table depicting the structural tongue abnormalities in various sites according to the age range of the study population.

Site	18-40 years n (%)	41-65 years n (%)	>65 years n (%)	p-value
Anterior 2/3 ^rd^	23 (3.4)	22 (5.3)	3 (6.4)	0.173
Posterior 1/3 ^rd^	13 (1.9)	11 (2.6)	1 (2.1)	0.614
Floor of mouth	4 (0.6)	17 (4.1)	0 (0)	<0.001*
Inferior surface of the tongue	1 (0.1)	2 (0.5)	0 (0)	0.613
Tip of the tongue	12 (1.8)	8 (1.9)	1 (2.1)	0.791
The lateral border of the tongue	8 (1.2)	15 (3.6)	1 (2.1)	0.024*

Functional abnormalities of tongue were noted in 8.7% of the study participants of which 3.7% had restricted tongue movement ( Table 4).

**Table 4.** Table depicting the frequency distribution of functional abnormalities of the tongue.

Functional abnormalities	Present (%)
Deviation	5 (0.4)
Taste dysfunction	53 (4.6)
Restricted tongue movement	42 (3.7)

The range of tongue protrusion observed in the study was 15 mm to 54 mm with a mean of 40.5 mm. Table 5 depicts the range of tongue protrusion in the study population.

**Table 5.** Range of tongue protrusion observed in the study population.

S. No	Distribution of tongue protrusion (mm)	Males	Females	Total (%)
1	50-60	07	15	22 (1.9)
2	40-50	266	278	544 (47.6)
3	30-40	245	258	503 (44)
4	20-30	30	28	58 (5.1)
5	10-20	16	0	16 (1.4)
6	0-9	0	0	0

Using Fischer's exact test, the association between tongue abnormalities and tobacco-associated habits was assessed. There was a positive association of tobacco-related habits with burning, pain, oral submucous fibrosis, verrucous carcinoma, squamous cell carcinoma, coated tongue, candidiasis, restricted tongue movement, and taste dysfunction ( Table 6).

**Table 6.** Association of smoking habit and lesions affecting the tongue.

Structural lesions	
		N (%)	N (%)	P-value
Burning	Absent	922 (94.5)	127 (76.0)	<0.001 *
	Present	54 (5.5)	40 (24.0)	
Pain	Absent	933 (95.6)	130 (77.8)	<0.001 *
	Present	43 (4.4)	37 (22.2)	
Coral Pink	Absent	76 (7.8)	17 (10.2)	0.296
	Present	900 (92.2)	150 (89.8)	
Red	Absent	921 (94.4)	155 (92.8)	0.431
	Present	55 (5.6)	12 (7.2)	
Pale	Absent	969 (99.3)	164 (98.2)	0.169
	Present	7 (0.7)	3 (1.8)	
Coarse tongue	Absent	73 (7.5)	26 (15.6)	0.001 *
	Present	903 (92.5)	141 (84.4)	
Smooth	Absent	915 (93.8)	147 (88.0)	0.008 *
	Present	61 (6.2)	20 (12.0)	
Fissured tongue	Absent	967 (99.1)	163 (97.6)	0.108
	Present	9 (0.9)	4 (2.4)	
Enlarged	Absent	965 (98.9)	142 (85.0)	<0.001 *
	Present	11 (1.1)	25 (15.0)	
Depapillation	Absent	923 (94.6)	148 (88.6)	0.003 *
	Present	53 (5.4)	19 (11.4)	
Coating on tongue	Absent	741 (75.9)	102 (61.1)	<0.001 *
	Present	235 (24.1)	65 (38.9)	
Ulcers	Absent	943 (96.6)	152 (91.0)	0.001 *
	Present	33 (3.4)	15 (9.0)	
Candidiasis	Absent	967 (99.1)	160 (95.8)	0.005 *
	Present	9 (0.9)	7 (4.2)	
Leukoplakia	Absent	973 (99.7)	165 (98.8)	0.157
	Present	3 (0.3)	2 (1.2)	
Lichen planus	Absent	965 (98.9)	160 (95.8)	0.01 *
	Present	11 (1.1)	7 (4.2)	
Oral Submucous fibrosis	Absent	972 (99.6)	147 (88.0)	<0.001 *
	Present	4 (0.4)	20 (12.0)	
Verrucous carcinoma	Absent	976 (100)	165 (98.8)	0.001 *
	Present	0 (0)	2 (1.2)	
Squamous Cell Carcinoma	Absent	976 (100)	163 (97.6)	<0.001 *
	Present	0 (0)	4 (2.4)	
Geographic tongue	Absent	964 (98.8)	163 (97.6)	0.274
	Present	12 (1.2)	4 (2.4)	
Pemphigus	Absent	973 (99.7)	167 (100)	0.473
	Present	3 (0.3)	0 (0)	
Erythroplakia	Absent	975 (99.9)	167 (100)	>0.99
	Present	1 (0.1)	0 (0)	
Erythema multiforme	Absent	974 (99.8)	165 (98.8)	0.104
	Present	2 (0.2)	2 (1.2)	
Allergy	Absent	971 (99.5)	163 (97.6)	0.031 *
	Present	5 (0.5)	4 (2.4)	
Erythematous candidiasis	Absent	975 (99.9)	166 (99.4)	0.271
	Present	1 (0.1)	1 (0.6)	
Varices	Absent	901 (92.3)	145 (86.8)	0.019 *
	Present	75 (7.7)	22 (13.2)	
Growth/Swelling	Absent	963 (98.7)	152 (91)	<0.001 *
	Present	13 (1.3)	15 (9.0)	
Deviation	Absent	974 (99.8)	164 (98.2)	0.024 *
	Present	2 (0.2)	3 (1.8)	
Restriction of tongue movement	Absent	963 (98.7)	138 (82.6)	<0.001 *
	Present	13 (1.3)	29 (17.4)	
Taste dysfunction	Absent	952 (97.5)	138 (82.6)	<0.001 *
	Present	24 (2.5)	29 (17.4)	

* Statistically significant.

## Discussion

Tongue abnormalities are commonly encountered in dental practice but often overlooked, if asymptomatic. The tongue may be affected by a wide range of local and systemic conditions. Structural and functional tongue abnormalities can be easily recognized on careful and detailed clinical examination. The present study evaluated the prevalence of structural and functional abnormalities of the tongue in a tertiary care hospital-based population. There is limited literature on the measurements of normal protrusive tongue movements. Understanding the physiologic range of tongue protrusion in a normal population aids in the early identification of tongue restriction. Restriction in the tongue protrusion occurs in various conditions like oral submucous fibrosis, carcinomas of the base of the tongue, scleroderma, and neurological disorders.

In the present study, overall tongue abnormalities were present in 66.5% of the subjects. A Turkish study by Avcu *et al.* (2003) showed a prevalence of tongue lesions in 52.2% of the study subjects. ^9^ Whereas, other studies from Turkey, India, and Libya showed a low prevalence of 4.9%, 2.8%, and 9.2%, respectively. ^10^^–^^12^

The anatomic distribution of tongue lesions was majorly noted on the anterior two-thirds of the tongue (4.2%) and least seen in the ventral surface of the tongue (0.3%). A study on the Libyan population showed that 90% of the lesions were on the dorsum, followed by the lateral border (7%), and posterior one-third (2%). The least common site was the ventral surface of the tongue (1%), which is in accordance with the present study. ^12^

The most prevalent comorbidity in the study population was diabetes mellitus (10.5%), followed by hypertension (8.9%). Bhattacharya *et al.* (2016) estimated the prevalence of diabetes mellitus to be 3.1%, which is lower than the observations from the current study. ^3^ A similar study on the Libyan population by Byahatti *et al.* (2010) found an even lower prevalence of subjects affected with diabetes mellitus (0.4%), followed by hypertension (0.3%). ^12^ Patil *et al.* (2013) conducted a study in which the prevalence of anemia was 3.8%, which was higher in comparison to the present study. ^13^

In the present study, 5.9% had a red erythematous tongue and 0.9% had a pale tongue. Physiologic melanin pigmentation of the tongue was noted in 4.8% of the study subjects, whereas other studies by Mumcu *et al.* (2005) ^14^ and Shinde *et al.* (2017) ^11^ were 6.9% and 2.1%, respectively. There is no literature available that has assessed the prevalence of other color variations on the tongue.

A positive oral abusive habit history was noted in 25.7% of the study population, of which 14.6% were tobacco users and 11.1% had alcohol consumption. In a study done by Motallebnejab *et al.* (2008), 54.7% of patients smoked tobacco and 35.3% had an alcohol habit. ^15^ Other studies had estimated the prevalence of tongue lesions in smokers to be 1.93%. ^12^ A positive association between smoking and the presence of hairy tongue was found by Darwazeh *et al.* (2010). ^16^

There was a positive association between tobacco-related habits and a burning sensation of the tongue in the present study. Tobacco-related habits were also associated with coated tongue, ulcers, increased incidence of candidiasis, oral lichen planus, oral submucous fibrosis, verrucous carcinoma, squamous cell carcinoma, restricted tongue movement, and taste dysfunction. Similarly, Bhattacharya *et al.* found a positive association between oral lichen planus and oral abusive habits. ^3^ Avcu *et al.* estimated the correlation between tongue lesions and smoking in the Turkish population and concluded that tongue lesions (hairy tongue, fissured tongue, and coated tongue) were increased in smokers and heavy smokers than in non-smokers. ^9^ Among the structural tongue abnormalities, the coated tongue was the most prevalent finding (26.2%), followed by depapillation (6.3%), ulcers (4.2%), enlarged tongue (3.1%), oral submucous fibrosis (2.5%) in the present study. Similarly, the coated tongue was the most prevalent finding (30.6%) followed by a fissured tongue (20.1%) ^3^ in a study conducted by Bhattacharya *et al.*, on the Indian population. The prevalence of coated tongue was 28.0% in an Indian study ^13^ by Patil S *et al*. Shinde *et al.* (2017) estimated the prevalence of fissured tongue (51.7%) was high, followed by coated tongue (14.3%), and geographic tongue (10%). ^11^ A higher prevalence of fissured tongue (48.4%) was observed in a study in the Libyan population. ^12^ By contrast, a very low prevalence of 1.1% with fissured tongue was noted in our study population. Lingual varicosity was noted in 8.5% of our subjects. Whereas, varicosity was the least common finding (0.4%) in an Indian population by Bhattacharya PT. ^3^

To the best of our knowledge, the present study was the first to evaluate the presence of functional abnormalities of the tongue that included restriction of tongue protrusion and taste dysfunction. No literature has addressed the various functional abnormalities that affect the tongue in a large population. Taste abnormalities were reported in 4.6% of the study population; 6.5% of the study subjects had tongue protrusion less than 30 mm. This is the first study that has evaluated the functional as well as structural abnormalities affecting the tongue. Kotlow *et al.* (1999) graded ankyloglossia into four classes: Class I- Mild (12-16 mm), Class II- Moderate (8-11 mm), Class III- Severe (3-7 mm), Class IV- Complete (less than 3 mm). ^17^ Considering the tongue protrusion range observed in the present study population and the classification of ankyloglossia, we propose a new grading for tongue protrusion, as depicted in Table 7.

**Table 7.** Proposed grading system of tongue protrusion inferred from the present study.

Grade 0	Normal tongue protrusion	Normal range 30-50 mm
Grade 1	Mild restriction of tongue protrusion	Tongue protrusion 20-29 mm
Grade 2	Moderate restriction of tongue protrusion	Tongue protrusion 10-19 mm
Grade 3	Severe restriction of tongue protrusion	Tongue protrusion <10 mm

Limitations of the present study include the observational nature due to which the contributing factors like oral hygiene, oral abusive habits, medications, and systemic disorders could not be associated with the findings on the tongue. Multicentric studies from diverse populations shall aid in a deeper understanding of the abnormalities that can affect the tongue.

## Conclusion

The results from the present study reflected a high prevalence of structural and functional abnormalities affecting the tongue. These abnormalities may be either clinically symptomatic or incidental findings on routine oral examination. A careful and detailed evaluation of the tongue examination is mandated to rule out any significant oral lesions. This shall help in the prompt identification of various etiological factors causing structural and functional abnormalities of the tongue. Understanding the normal tongue protrusion range will aid in the early detection of restricted tongue movements.

## Data Availability

Mendeley Data: Structural and functional abnormalities of the tongue: An epidemiological study from a tertiary center with a proposed grading system for tongue protrusion. https://doi.org/10.17632/k3vx49rbfn.1. ^18^ Data are available under the terms of the Creative Commons Attribution 4.0 International license (CC-BY 4.0).
