# Supplementary material for: Structural and functional abnormalities of the tongue: An epidemiological study from a tertiary care center in India
Source: F1000Res. 2024 Feb 16;12:822. Originally published 2023 Jul 13. [Version 2] doi: 10.12688/f1000research.131661.2 (PMC10915362; doi:10.12688/f1000research.131661.2)
Supplement: Peer review history

# Reviewer report: Reviewer response for version 2

**DOI:** 10.5256/f1000research.161405.r247887

**Published:** 2024-03-06

**Authors:** Bijimole Jose

**Affiliations:** 1 Pediatric Dentistry, Al Azhar Dental College, Thodupuzha, Kerala, India

No further comments.

Is the work clearly and accurately presented and does it cite the current literature?

Yes

If applicable, is the statistical analysis and its interpretation appropriate?

Yes

Are all the source data underlying the results available to ensure full reproducibility?

Yes

Is the study design appropriate and is the work technically sound?

Yes

Are the conclusions drawn adequately supported by the results?

Yes

Are sufficient details of methods and analysis provided to allow replication by others?

Yes

Reviewer Expertise:

dentistry

I confirm that I have read this submission and believe that I have an appropriate level of expertise to confirm that it is of an acceptable scientific standard.

# Reviewer report: Reviewer response for version 2

**DOI:** 10.5256/f1000research.161405.r247888

**Published:** 2024-03-05

**Authors:** Karpal Singh Sohal

**Affiliations:** 1 Oral and Maxillofacial Surgery, Muhimbili National Hospital and Muhimbili University of Health and Allied Sciences, Dar es Salaam, Tanzania

Report: 

 The authors have done significant improvements in their work, though there are minor grammatical errors, the main concerns that I had raised earlier have been taken care of.

 Status:

 I approve the revised version.

Is the work clearly and accurately presented and does it cite the current literature?

Partly

If applicable, is the statistical analysis and its interpretation appropriate?

Yes

Are all the source data underlying the results available to ensure full reproducibility?

Yes

Is the study design appropriate and is the work technically sound?

Partly

Are the conclusions drawn adequately supported by the results?

Partly

Are sufficient details of methods and analysis provided to allow replication by others?

No

Reviewer Expertise:

oral and maxillofacial surgery, oral medicine, head and neck oncology, clinical dentistry, oral and maxillofacial radiology

I confirm that I have read this submission and believe that I have an appropriate level of expertise to confirm that it is of an acceptable scientific standard.

# Reviewer report: Reviewer response for version 1

**DOI:** 10.5256/f1000research.144523.r228869

**Published:** 2024-01-10

**Authors:** Karpal Singh Sohal

**Affiliations:** 1 Oral and Maxillofacial Surgery, Muhimbili National Hospital and Muhimbili University of Health and Allied Sciences, Dar es Salaam, Tanzania

**Reviewer of Manuscript titled: Structural and Functional Abnormalities of the Tongue: An Epidemiological Study from a Tertiary Care Center.**

**General Comments**

 This manuscript was meant to report on the incidence and pattern of structural and functional abnormalities of the tongue.

*Overall Assessment of the Manuscript*

 First and foremost the authors are congratulated for coming up with such an interesting study. The authors have collected valuable data. There are some flaws in the manuscript as described in section wise comments.

**Section-wise comments**

**The Title:       **

1. The title length is acceptable.
2. The title is specific and concise.

***Recommendation: it would have been better if the title could describe the population studied.***

**Abstract:        **

1. It is according to the standards of the journal.
2. The aim of the article has been stated.
3. In the methods section, the authors summarize the necessary details.
4. The main result has been described.
5. Conclusion: is out of the context, the aim is to report on structural and functional abnormalities, and not merely lesions of the tongue
6. Few grammatical errors.

***Recommendation: there is room for improving the grammar used and the conclusion***

**Keywords:  **

1. The number of Keywords is acceptable.
2. Keywords are not appropriate.

***Recommendation: Please re-write the keywords.***

**Introduction: **

1. The introduction fails to describe the subject at hand adequately, it derails the reader from the main purpose of the study
2. The rationale for carrying out this study is stated.
3. The objective is not apparent
4. A few grammatical errors were noted.
5. The knowledge gap that the authors are trying to address is apparent.
6. A clear flow of thoughts between paragraphs and in some places between sentences within the same text is lacking. The authors have repeated some words and ideas within the same paragraph.
7. Citation is appropriate

***Recommendation: Please re-phrase the introduction.  In the introduction section, the authors may add some literature review regarding the subject at hand.***

**Methods:**

  

1. The study design is clearly described.
2. The population studied is not known. Though the authors report that the study was carried out in Manipal, it can not be assumed that the individuals recruited were Indians.
3. Inclusion and exclusion criteria are very narrow. E.g Did the authors include patients with mental challenges, or those presenting with orofacial space infections?
4. How many clinicians examined all the patients?
5. How were inter- and intra-observer reliability taken care of?
6. The statistical analysis carried out has been briefly described
7. Few grammatical errors were noted.

***Recommendation: Please improve the methodology section.***

**Results:          **

1. The results have been presented fairly and in a very simplified manned
2. *Few grammatical errors.*

***Recommendation: there is room for improving the results. Considering the vast data authors have collected, it would make sense if the authors could compare the abnormalities by age group and sex, and the structural abnormalities by the site.***

**Discussion:     **

1. The authors have tried to discuss the results by basically comparing their results to those of other studies with minimum explanation and interpretation of the similarities and differences noted.
2. There is repetition in reporting the results in the discussion section
3. The flow of information requires improvements.
4. The authors have discussed some of the limitations.
5. The authors have disclosed the importance of the research findings to the community and the practice in brief.
6. Significant grammatical errors.

***Recommendation: There is room for some improvement.***

**Conclusion:    **

1. The conclusion does not reflect the objectives of the study.

***Recommendation: Please re-read the conclusion***

**References and citations:     **

1. References are written as per journal guidelines.
2. The number of references is acceptable.

***Recommendation: None.***

**Structure and length:           **

1. It is a lengthy paper.
2. The article is organized and balanced.
3. It has relevant information regarding a tongue abnormalities in x-population.

***Recommendation: None***

**Tables :          **

1. An acceptable number of tables.
2. The tables are relevant.

***Recommendation: none***

**English:          **

1. The English used in the article is acceptable.
2. There are a few obvious grammatical mistakes.

***Recommendation: there is room for improving the language***

**Logic:             **

1. The article has been written clearly except for the introduction part.
2. There is a minimum violation of grammar in the manuscript, however, it does not hinder the reader from grabbing information easily.

***Recommendation: There is room for improvements.***

Is the work clearly and accurately presented and does it cite the current literature?

Partly

If applicable, is the statistical analysis and its interpretation appropriate?

Yes

Are all the source data underlying the results available to ensure full reproducibility?

Yes

Is the study design appropriate and is the work technically sound?

Partly

Are the conclusions drawn adequately supported by the results?

Partly

Are sufficient details of methods and analysis provided to allow replication by others?

No

Reviewer Expertise:

oral and maxillofacial surgery, oral medicine, head and neck oncology, clinical dentistry, oral and maxillofacial radiology

I confirm that I have read this submission and believe that I have an appropriate level of expertise to confirm that it is of an acceptable scientific standard, however I have significant reservations, as outlined above.

# Reviewer report: Reviewer response for version 1

**DOI:** 10.5256/f1000research.144523.r222602

**Published:** 2023-12-15

**Authors:** Bijimole Jose

**Affiliations:** 1 Pediatric Dentistry, Al Azhar Dental College, Thodupuzha, Kerala, India

An interesting and relevant topic is discussed in this article. Studies on functional abnormalities of the tongue are rare in literature. Researchers included all the relevant tongue lesions and functional abnormalities in this study.

 comments:

 1.Table 1- macroglossia is size variation.

 2.Table 2- add restriction of tongue movements and its  percentage 

 3. Abstract; result- Prevalence of functional abnormalities can be added.

 Article can be accepted with these minor corrections.

Is the work clearly and accurately presented and does it cite the current literature?

Yes

If applicable, is the statistical analysis and its interpretation appropriate?

Yes

Are all the source data underlying the results available to ensure full reproducibility?

Yes

Is the study design appropriate and is the work technically sound?

Yes

Are the conclusions drawn adequately supported by the results?

Yes

Are sufficient details of methods and analysis provided to allow replication by others?

Yes

Reviewer Expertise:

dentistry

I confirm that I have read this submission and believe that I have an appropriate level of expertise to confirm that it is of an acceptable scientific standard.
